# Cytokine-Induced Killer Cell Immunotherapy Reduces Recurrence in Patients with Early-Stage Hepatocellular Carcinoma

**DOI:** 10.3390/cancers17040566

**Published:** 2025-02-07

**Authors:** Dong Hyun Kim, Eun Min Kim, Jae Seung Lee, Mi Na Kim, Beom Kyung Kim, Seung Up Kim, Jun Yong Park, Gi Hong Choi, Sang Hoon Ahn, Hye Won Lee, Do Young Kim

**Affiliations:** 1 Department of Internal Medicine, Yonsei University College of Medicine, Seoul 03722, Republic of Korea; chefcurry2175@yonsei.ac.kr (D.H.K.); trucamp7@yonsei.ac.kr (E.M.K.); sikarue@yuhs.ac (J.S.L.); minakim@yuhs.ac (M.N.K.); beomkkim@yuhs.ac (B.K.K.); ksukorea@yuhs.ac (S.U.K.); drpjy@yuhs.ac (J.Y.P.); ahnsh@yuhs.ac (S.H.A.); 2 Institute of Gastroenterology, Yonsei University College of Medicine, Seoul 03722, Republic of Korea; 3 Yonsei Liver Center, Severance Hospital, Seoul 03722, Republic of Korea; 4 Department of Surgery, Yonsei University College of Medicine, Seoul 03722, Republic of Korea; choigh@yuhs.ac

**Keywords:** hepatocellular carcinoma, cytokine-induced killer cell, recurrence, immunotherapy, tumor marker

## Abstract

Background/Objectives: Cytokine-induced killer (CIK) cell immunotherapy has shown promise in reducing recurrence and improving survival outcomes in hepatocellular carcinoma (HCC). We evaluated the efficacy and safety of CIK cell therapy in a real-world clinical setting. Methods: A retrospective analysis was conducted on 49 patients who received CIK cell therapy after curative resection or radiofrequency ablation, compared with 49 matched control patients via 1:1 propensity score matching. The primary endpoint was recurrence-free survival (RFS), and the secondary endpoint was overall survival (OS). Results: The median follow-up durations were 19.1 months for the immune cell group and 67.7 months for the control group. In univariable analysis, the immune cell group demonstrated a prolonged RFS than the control group (hazard ratio [HR], 0.32; 95% CI, 0.15–0.71; log-rank *p* = 0.001). The median RFS was not reached in the immune cell group but was 48.62 months in the control group. A multivariable Cox regression model identified CIK cell therapy as a significant factor associated with a reduced risk of HCC recurrence (adjusted HR, 0.32; 95% CI, 0.15–0.71; *p* = 0.005). The median OS was not reached in either group; no significant differences in OS were observed between the immune cell and control groups (log-rank *p* = 0.082). The overall incidence of adverse events was low, and no Grade 3 or 4 events were reported. Conclusions: Adjuvant CIK cell immunotherapy after curative treatment significantly prolongs RFS in early-stage HCC patients. Further research regarding the broader applications of CIK cell immunotherapy in HCC is warranted.

## Simple Summary

This study evaluates the effectiveness of cytokine-induced killer (CIK) cell therapy as an adjuvant treatment for patients with early-stage liver cancer (HCC). CIK therapy significantly lowered the risk of recurrence by 68% and reduced key tumor marker levels in patients with elevated markers as well, with no severe adverse events. Our results highlight the potential of CIK therapy to prevent early recurrence and support its broader clinical application.

## 1. Introduction

Hepatocellular carcinoma (HCC) is the most common type of primary liver cancer and a leading cause of cancer-related deaths worldwide, predominantly arising in patients with chronic liver disease and cirrhosis [1]. Risk factors include hepatitis B and C infections, alcohol consumption, and metabolic dysfunction-associated steatotic liver disease [2]. HCC constitutes a substantial health burden, particularly in regions with a high prevalence of hepatitis B and C infections, such as East Asian nations, including Korea [3]. The incidence and prevalence of HCC continue to rise, paralleling the global increases in risk factors such as metabolic syndrome and viral hepatitis persistence [4]. Chronic liver injury from these risk factors leads to cycles of hepatocyte damage, inflammation, and regeneration, resulting in fibrosis and cirrhosis, which create a tumor-promoting environment [5,6]. For early-stage HCC, curative options such as surgical resection or radiofrequency ablation (RFA) are commonly employed [7,8].

Despite advancements in curative treatments, up to 70% of HCC patients experience recurrence within five years, a rate significantly higher than that of other major cancers (e.g., early gastric and colorectal cancers) [9,10,11]. Early diagnosis through active surveillance is crucial for efforts to improve outcomes because it allows timely intervention when tumors are more treatable [12,13]. However, the frequent diagnosis of HCC at advanced stages, combined with its high recurrence rate, highlights the need for more effective adjuvant therapies to further enhance long-term survival outcomes [14]. The tumor microenvironment promotes immune evasion by inhibiting cytotoxic T-cell responses and increasing the activity of regulatory T cells and myeloid-derived suppressor cells, posing substantial challenges for effective therapy [15]. Therefore, personalized treatment strategies, which consider individual risk factors and tumor biology, and novel adjuvant options are essential to address distinct recurrence patterns.

**Figure 1:**
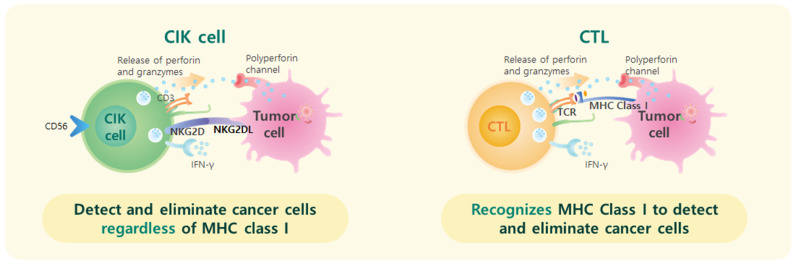
Comparison of MHC-independent and MHC-dependent tumor killing mechanisms by CIK cells and CTLs. CIK cells exhibit a unique MHC-independent tumor-specific killing mechanism, unlike CTLs, which rely on MHC for tumor recognition. This highlights the therapeutic potential of CIK cells, particularly in cancers with low or absent MHC expression, such as HCC. Abbreviations: CIK, cytokine-induced killer; CTL, cytotoxic T lymphocyte; MHC, major histocompatibility complex; IFN, interferon; NK, natural killer; TCR, T-cell receptor.

Recently, the exploration of immunotherapeutic approaches has opened new avenues for potential interventions in malignancies. Among these, cytokine-induced killer (CIK) cells have emerged as a promising option. CIK cells are a heterogeneous subset of immune cells characterized by the expression of both T-cell and natural killer (NK) cell markers, particularly CD3+/CD56+. These cells exhibit potent cytotoxic activity against tumor cells in a non-major histocompatibility complex (MHC)-restricted manner, which makes them suitable for adoptive immunotherapy in cancer treatment [16]. Currently, CIK cells are investigated and used for the treatment of various types of cancers, including HCC, colorectal cancer, gastric cancer, and non-small cell lung cancer (NSCLC) [17]. Their abilities to target and kill tumor cells regardless of MHC expression enhance their potential effectiveness in treating diverse cancers, as shown in Figure 1 [18].

Recent studies have demonstrated the potential for CIK cell therapy to reduce HCC recurrence and improve survival outcomes in controlled trial settings and real-world clinical practice [19,20,21]. CIK cells from HCC patients reveal elevated NK (DNAM1, NKG2D) and chemokine (CXCR3, CD62L) receptors for tumor-site trafficking, and low immune checkpoint expression (PD-1, CTLA-4, LAG-3) to minimize immune suppression [22]. CIK cell therapy, classified as an A-level recommendation for early-stage HCC in Korean guidelines, is expected to see improved patient access with its inclusion in national insurance coverage alongside other antitumor agents [23]. Previous studies on CIK cell therapy in Korea, while informative, were conducted before the introduction of the 2022 practice guidelines [24,25]. Thus, there is a need for further research to assess CIK cell therapy within the context of the most recent guidelines.

This study evaluated the efficacy and safety of CIK cell therapy as an adjuvant treatment for HCC patients in the context of the updated 2022 guidelines. We sought to provide a more comprehensive and up-to-date perspective on the viability of CIK cell therapy as a standard treatment option for HCC.

## 2. Materials and Methods

### 2.1. Study Population

This retrospective study included patients with HCC who underwent curative treatments, including surgical resection or RFA at Severance Hospital [Seoul/Korea] between January 2012 and March 2024. The study population was divided into two groups: the immune cell group, consisting of patients who received adjuvant CIK cell therapy, and the control group, including patients who did not receive any adjuvant treatment. Eligibility criteria included patients aged >19 years with confirmed HCC based on histological examination or radiological imaging according to the American Association for the Study of Liver Diseases guidelines and Korean Liver Cancer Association (KLCA) and National Cancer Center (NCC) Korea practice guidelines [23,26]. Patients were required to have a radiological diagnosis of stage I or II HCC based on the American Joint Committee on Cancer (AJCC) Staging Manual (6th edition), along with Child-Pugh class A liver function and an Eastern Cooperative Oncology Group performance status of 0 or 1.

**Figure 2:**
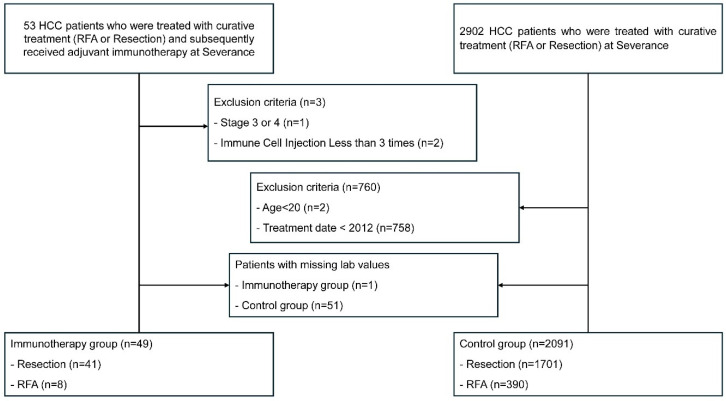
Flow chart of study population. The initial dataset included 53 HCC patients who received RFA or resection as curative therapy and subsequently received immune cell therapy as adjuvant therapy, as well as 2902 patients who did not receive any adjuvant therapy. This retrospective study applied the following exclusion criteria: patients younger than 20 years, treatment dates before 2012, and those with stage III or IV cancer. Additionally, for the immune cell group, patients who received immune cell injection less than 3. Finally, patients with missing lab values were excluded as well. Abbreviations: HCC, hepatocellular carcinoma; RFA, radiofrequency ablation.

Patients were excluded if they had advanced HCC (stage III/IV, n = 1) per 2022 KLCA-NCC guidelines recommending CIK therapy only for early-stage HCC, received fewer than three CIK injections (n = 2) to ensure sufficient therapy exposure, were younger than 20 years (n = 2), had treatments before 2012 (n = 758) to align with the immune cell group, which began treatments in 2012, or had missing laboratory data (n = 52), as shown in Figure 2.

### 2.2. Study Design and Treatment Protocol

To prepare CIK cells, 150 mL of peripheral blood was drawn from each patient in the immune cell group approximately 2–3 weeks prior to the start of cell therapy. Peripheral blood mononuclear cells (PBMCs) were isolated from whole blood through density gradient centrifugation and expanded for 12–21 days using interleukin (IL)-2 and anti-CD3 monoclonal antibody, in accordance with a published protocol [25]. The prepared CIK cell agent (200 mL) was administered intravenously within 60 min. Patients were monitored for a minimum of 30 min post-infusion to assess any immediate adverse reactions. The CIK cell agent (Immuncell-LC; Green Cross Cell Corp, Seoul, Republic of Korea) was administered in a total of 16 doses over 59 weeks, following a structured schedule: four infusions weekly, followed by four biweekly, four every four weeks, and the final four every eight weeks. Treatment was discontinued upon detection of HCC recurrence. This study adhered to the Declaration of Helsinki and was approved by the institutional review board of Severance Hospital (IRB No. 4-2020-1081, 16 November 2020).

### 2.3. Endpoints and Treatment Evaluation

The primary endpoint of the study was recurrence-free survival (RFS), defined as the time from the date of curative treatment to the date of HCC recurrence or last follow-up. Overall survival (OS), defined as the time from the date of curative treatment to the date of death from any cause or last follow-up, was considered the secondary endpoint. Safety assessments included the incidence and severity of adverse events, graded according to the Common Terminology Criteria for Adverse Events, Version 5.0. Adverse events were monitored from the start of CIK cell therapy until the study end or patient dropout. Treatment evaluations were performed using dynamic computed tomography (CT) or magnetic resonance imaging (MRI), with tumor markers including alpha-fetoprotein (AFP) and protein induced by vitamin K antagonist-II (PIVKA-II), 1 month after the first treatment and every 3–6 months thereafter.

To further assess treatment efficacy, patients were categorized into high and low tumor marker level groups based on post-treatment AFP (>10 ng/mL) and PIVKA-II (>40 mAU/mL) levels [27,28]. Those with AFP ≤ 10 ng/mL or PIVKA-II ≤ 40 mAU/mL were classified as low tumor marker level group. Changes in AFP and PIVKA-II levels before and after immunotherapy were analyzed to assess treatment effects on tumor marker levels.

To ensure that post-treatment marker levels accurately reflect residual HCC activity rather than transient changes due to hepatocyte regeneration, we set the timing for marker measurement at 1 month or more after treatment. Serum AFP and PIVKA-II levels may transiently increase due to liver regeneration following curative treatments, typically normalizing within 2–3 weeks [29,30,31,32,33]. By measuring marker levels at least 1-month post-treatment, we minimized the influence of regeneration-related fluctuations and focused on persistent marker elevations.

### 2.4. Statistical Analyses

To minimize selection bias arising from imbalanced baseline characteristics between the immunotherapy and control groups, propensity score (PS) matching was applied using a 1:1 nearest-neighbor matching algorithm without replacement [34]. Each patient’s PS was derived using a multivariable logistic regression model. Standardized mean differences (SMDs) were used to assess the balance of variables between the two groups; an absolute value of less than 0.30 indicated good balance [35]. Data are presented as median (interquartile range [IQR]) or as counts with percentages (n [%]). Differences between groups were analyzed using the Mann–Whitney U test for continuous variables and the chi-square or Fisher’s exact test for categorical variables. Kaplan–Meier method was utilized to generate survival curves for RFS and OS, with group comparisons performed using the log-rank test. Crude hazard ratios (HRs) were calculated using a Cox proportional hazards model. A forest plot was created to illustrate subgroup analyses, comparing the impact of immunotherapy on RFS between the immune cell and control groups. Using variables that showed significant associations in univariable analysis, as well as a stepwise forward selection method, multivariable Cox regression model was applied to determine key predictors of RFS. Additional analyses were performed focusing on the immune cell group, specifically those with tumor marker values available before and after curative treatment and immune cell injection. All tests were two-sided, and *p*-values < 0.05 were considered statistically significant. PS matching was performed using the Python package PsmPy (version 0.3.13; Python Software Foundation, Beaverton, OR, USA, 2024). All other analyses were conducted using R software (version 4.4.1; R Core Team, Vienna, Austria, 2024).

## 3. Results

### 3.1. Patients

Between January 2012 and March 2024, a total of 2140 HCC patients who received surgical resection or RFA at Severance Hospital met the inclusion criteria for this study. A total of 49 patients were allocated to the immune cell group (41 resections, 8 RFA) and 2091 patients were assigned to the control group (1701 resections, 390 RFA). All patients included in the study were classified as AJCC stage T1N0M0. The median follow-up durations were 19.1 months (IQR: 11.3–49.9 months) in the immune cell group and 67.7 months (IQR: 36.8–103.4 months) in the control group.

Before PS matching, significant differences were observed in the frequency of fatty liver (*p* < 0.001), PIVKA-II levels (*p* = 0.021), and serum levels of albumin (*p* = 0.003) between the groups. PS matching was conducted using a 1:1 nearest-neighbor algorithm, adjusted for variables such as age, sex, treatment type, liver cirrhosis, fatty liver, total bilirubin, aspartate aminotransferase, and platelet count. After matching, the differences in fatty liver (*p* = 0.809, SMD = 0.098), PIVKA-II levels (*p* = 0.696, SMD = 0.285), and albumin levels (*p* = 0.136, SMD = 0.213) were no longer statistically significant. All other variables had *p*-values above 0.05 and SMD values below 0.30, as detailed in Table 1. Finally, 49 patients each from the immune cell and control groups were included in statistical analyses. The tumor characteristics, such as tumor size, grade, and presence of various types of invasion, showed no significant differences between the final immune cell group and the control group, as presented in Table S1.

Among 42 patients in the immune cell group (40 hepatitis B, 2 hepatitis C), 36 received anti-viral treatment (35 hepatitis B, 1 hepatitis C), while 35 of 41 control group patients (all hepatitis B) received anti-viral treatment. Fisher’s Exact Test showed no significant difference in anti-viral treatment rates between groups (*p* = 1.000). For hepatitis B patients, treatments included entecavir, tenofovir alafenamide, tenefovir disoproxil fumarate, lamivudine, and adefovir dipivoxil. The sole hepatitis C treatment was pegylated interferon α with ribavirin. Twelve patients did not receive anti-viral therapy due to undetectable hepatitis B virus DNA, hepatitis B surface antigen conversion, or resolved infection. Detailed anti-viral regimens are shown in Table S2.

### 3.2. Recurrence-Free Survival

The median RFS in the immune cell group was not reached; in the control group, it was 48.62 months. At the end of the study, 41 of 49 patients (83.7%) in the immune cell group remained free of recurrence or death, compared with 21 of 49 patients (42.9%) in the control group. The immune cell group exhibited a significantly lower risk of recurrence or death compared with the control group in univariable analysis (HR = 0.32; 95% confidence interval [CI]: 0.15–0.71; log-rank *p* = 0.001). The RFS rates at 24, 36, 60, 72, and 84 months were significantly higher in the immune cell group than in the control group; all one-sided z-test *p*-values were <0.05. At the 5-year mark, the RFS rates were 67.0% in the immune cell group and 47.5% in the control group, as detailed in Table S3.

**Figure 3:**
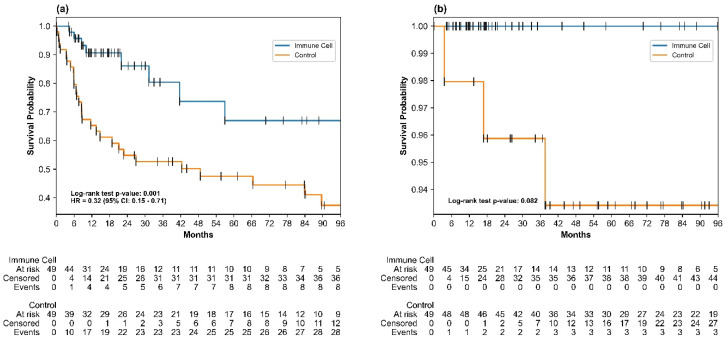
Kaplan–Meier plots for (**a**) recurrence-free survival and (**b**) overall survival. The frequency of recurrence observed by group is as follows: In the control group, 26 individuals (53.1%) did not experience an event, while 23 individuals (46.9%) did. In the immunotherapy group, 41 individuals (83.7%) did not experience an event, while 8 individuals (16.3%) did. The frequency of death by group is as follows: In the control group, 40 individuals (81.6%) were alive, while 9 individuals (18.4%) had died. In the immunotherapy group, 50 individuals (100.0%) were alive, while no deaths were observed. The ticks in the plot represent either an event or a cutoff. The log-rank *p*-value for RFS was 0.001, and for OS it was 0.082. The trend in the plot aligns with previous studies showing immune cell’s beneficial effect on reducing early recurrence (≤2 years) but not significantly with late recurrence (>2 years). Abbreviations: HR, hazard ratio; RFS, recurrence-free survival; OS, overall survival.

**Figure 4:**
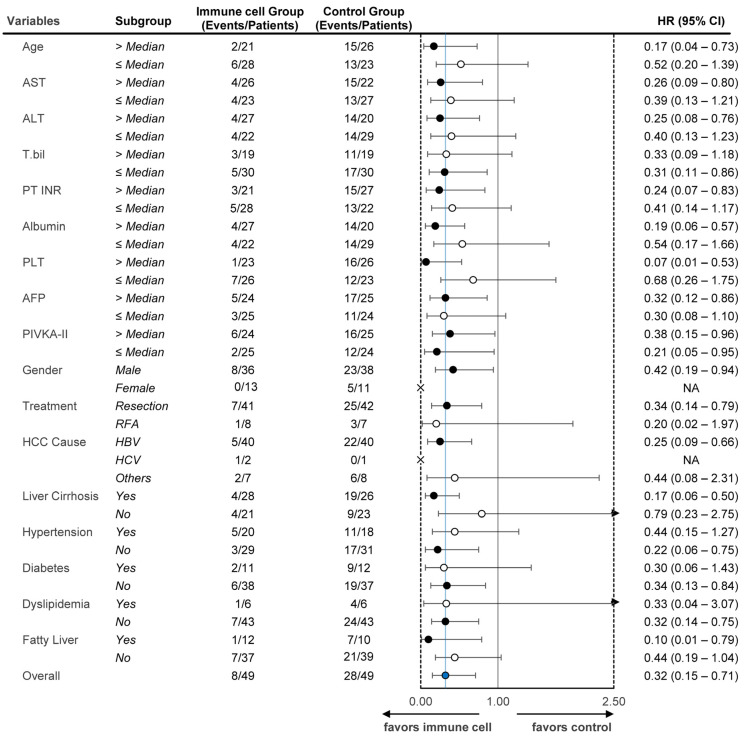
Subgroup analyses on recurrence-free survival. Subgroup analyses based on baseline characteristics are presented. In the sex variable, the female subgroup of the immune cell group had no recurrences. All subgroups had an HR below 1, indicating a consistent superiority of the immune cell group compared to the control group. Dots are filled black for subgroups with a statistically significant HR, and white if not significant. The arrow indicates whether the upper threshold exceeds 2.50. Abbreviations: AST, aspartate aminotransferase; ALT, alanine aminotransferase; T.bil, total bilirubin; PT INR, prothrombin time international normalized ratio; PLT, platelet count; AFP, alpha-fetoprotein; PIVKA-II, protein induced by vitamin K absence-II; RFA, radiofrequency ablation; HBV, hepatitis B virus; HCV, hepatitis C virus; NA, not applicable; CI, confidence interval; HR, hazard ratio. Blue dots reflect variables favoring immunce cell.

In the multivariable Cox proportional hazards analysis, after adjustment for key variables including PIVKA-II levels, the immune cell group continued to show a statistically significant improvement in RFS compared with the control group (adjusted HR = 0.32, 95% CI: 0.15–0.71, *p* = 0.005), as detailed in Table 2. Kaplan–Meier survival curves further confirmed the prolonged RFS in the immune cell group compared with the control group, as illustrated in Figure 3a. The log-rank test confirmed a significant difference in early recurrence (within 2 years; *p* < 0.001) but not in late recurrence (beyond 2 years; *p* = 0.834). Subgroup analyses across different baseline characteristics (e.g., liver cirrhosis and treatment type) all demonstrated HRs less than 1, as shown in Figure 4.

### 3.3. Overall Survival

As of the data cut-off, no deaths were reported in the immune cell group, whereas three deaths (6.1%) occurred in the control group. The majority of patients in both groups were still alive at the time of analysis: 46 of 49 (93.9%) in the control group and 49 of 49 (100%) in the immune cell group. The three deaths in the control group were attributed to liver failure due to HCC progression. Median OS values were not reached in either group; no statistically significant difference in OS was observed between the two groups (log-rank *p* = 0.082), as shown in Figure 3b.

### 3.4. Safety

No Grade 3 or 4 adverse events were reported in the immune cell group. Three minor adverse events were observed (6.1%): back pain, urticaria, and lower body edema. These events were determined to be unrelated to the immunotherapy, and all adverse events resolved naturally with conservative management. No infections or allergic reactions were reported.

### 3.5. Changes in Tumor Markers

In the high-tumor-marker group divided by post-treatment AFP levels (>10 ng/mL), the median AFP levels significantly decreased from 15.3 ng/mL to 1.3 ng/mL after treatment (*p* = 0.002), while PIVKA-II levels showed no significant change (*p* = 0.307). Conversely, in the low-tumor-marker group (post-treatment AFP ≤ 10 ng/mL), AFP levels decreased slightly from 2.8 ng/mL to 2.1 ng/mL, but this change was not statistically significant (*p* = 0.552). PIVKA-II levels also showed no significant difference, decreasing from 31.5 mAU/mL to 29.5 mAU/mL (*p* = 0.097), as shown in Table 3a.

When dividing by post-treatment PIVKA-II levels (>40 mAU/mL), the high-tumor-marker group exhibited a non-significant decrease in AFP levels from 10.4 ng/mL to 2.4 ng/mL (*p* = 0.084), whereas PIVKA-II levels significantly decreased from 42.0 mAU/mL to 27.0 mAU/mL (*p* = 0.019). In the low-tumor-marker group (post-treatment PIVKA-II ≤ 40 mAU/mL), AFP levels significantly decreased from 3.0 ng/mL to 1.5 ng/mL (*p* = 0.001), but PIVKA-II levels remained relatively unchanged, increasing slightly from 25.0 mAU/mL to 28.0 mAU/mL (*p* = 0.932), as shown in Table 3b. The overall changes in AFP and PIVKA-II levels for the entire cohort, without subgrouping according to tumor marker, are presented in Table S4.

## 4. Discussion

This study evaluated the efficacy and safety of CIK cell therapy as an adjuvant treatment for HCC patients who underwent curative resection or RFA. The results demonstrated a significant improvement in RFS for the immune cell group relative to the control group, with an adjusted HR of 0.32 (*p* = 0.005), indicating a 68% lower risk of recurrence or death. Univariable analysis and subgroup analyses further validated the benefit of immunotherapy across different patient characteristics, consistently demonstrating a hazard ratio of less than 1, indicating a favorable outcome in the immune cell group. The RFS rates at 24, 36, 60, 72, and 84 months were significantly higher in the immune cell group than in the control group. Early recurrence showed a highly significant difference between the two groups (*p* < 0.001), whereas late recurrence did not exhibit statistical significance (*p* = 0.834). Additionally, no Grade 3 or 4 adverse events were observed, and the minor adverse events reported were self-limiting. Tumor marker analysis indicated a significant decrease in the marker used to distinguish high and low tumor marker groups, with AFP levels significantly decreasing in the high AFP marker group and PIVKA-II levels in the high PIVKA-II marker group.

Our study is the first to be conducted following the 2022 update to the KLCA-NCC Korea practice guidelines, offering a real-world assessment of CIK cell therapy in early-stage HCC patients. Although CIK therapy for HCC is included in the Korean guidelines, economic barriers remain a challenge for many non-insurance and novel anticancer agents. As a result, national and societal strategies to enhance patient accessibility are actively under discussion [36,37]. Furthermore, despite its inclusion in the Korean context, CIK cell therapy has yet to be incorporated into international standard treatment protocols, largely due to the highly controlled nature of randomized controlled trials and lack of real-world evidence [38,39,40]. Thus, this study was conducted to obtain up-to-date, real-world data on the effectiveness of CIK cell therapy, helping to bridge the gap in evidence necessary to support its wider clinical adoption.

CIK cells are a distinct subset of T lymphocytes generated ex vivo by the stimulation of a patient’s PBMCs with anti-CD3 antibodies and IL-2; they are notable for MHC-independent cytotoxicity [41]. These cells exhibit both T-cell and NK cell characteristics, enabling them to recognize and destroy tumor cells without requiring prior sensitization or MHC restriction [42]. This property allows CIK cells to circumvent common immune evasion mechanisms, such as the loss or downregulation of the peptide-MHC-I molecules on cancer cell surfaces, which impair T cell immunorecognition [43]. Additionally, interactions between tumor cells and immune-suppressive cells within the tumor microenvironment impair the function of key effector cells such as cytotoxic T cells and dendritic cells [44]. CIK cells, particularly the CD3+/CD56+ subset, offer a potent solution for these limitations due to their enhanced cytotoxicity and ability to act independently of MHC restriction. They are enriched with CD8+ cells and granzyme content, enabling them to mediate more effective tumor cell lysis [45,46].

Previous adjuvant therapies, such as sorafenib, failed to show efficacy, while recent immunotherapy combinations, such as atezolizumab with bevacizumab, faced safety and efficacy challenges [9,14]. Ongoing studies on immune checkpoint inhibitors, including nivolumab, pembrolizumab, and durvalumab with bevacizumab, highlight concerns about limited efficacy in the absence of a robust tumor microenvironment and the risk of adverse events as well, such as gastrointestinal bleeding [47].

Our findings align with previous studies that have demonstrated the efficacy and safety of CIK cells as adjuvant therapy following curative treatment. The HR for RFS was 0.32, substantially below the range observed in previous research (0.59–0.67), suggesting greater effectiveness in our cohort [48,49]. This difference could be attributed to the fact that our immune cell group comprised 40 B-virus HCC patients and only nine non-B cases. Although many trials have demonstrated the efficacy of CIK therapy for B-virus, its effects on non-B causes remain less studied; there is a need for future research with a larger cohort of non-B patients [50,51]. The total incidence of adverse events in the immune cell group was 6.1%, with no severe complications observed. This low incidence may be attributed to the properties of CIK cells, which are derived from autologous PBMCs cultured ex vivo and stimulated with cytokines, minimizing toxicity and preventing graft-versus-host reactions [52,53]. Consistent with prior research, CIK cell therapy proved more effective for preventing early recurrence than late recurrence, likely because early recurrence is closely linked to the metastasis of residual neoplastic cells, rather than de novo hepatocarcinogenesis [54]. CIK cells, with their MHC-unrestricted cytotoxicity, are particularly suitable for the identification and clearance of these remaining malignant cells prior to proliferation. Given that early recurrence poses a higher risk than late recurrence for patients with HCC, targeting this period is particularly critical [55].

Additionally, our research demonstrates the effectiveness of CIK cell therapy in early-stage HCC and in patients with high tumor markers post-treatment. Considering that AFP and PIVKA-II are well-established biomarkers for predicting HCC recurrence, we focused on these markers in our analysis [56,57]. Tumor marker analysis indicated a significant decrease in AFP levels in the high AFP marker group and PIVKA-II levels in the high PIVKA-II marker group, highlighting the potential effectiveness of CIK therapy in high-risk patients. Our results, together with those of recent studies exploring strategies to enhance CIK therapy—including its combination with immune checkpoint inhibitors, the use of agents to stimulate MHC expression for better immune activation, and the downregulation of immune suppressor cells—underscore the potential applications of CIK beyond early-stage patients [58,59,60,61]. These findings suggest that CIK therapy could be extended to patients with high tumor markers, broadening its application to more aggressive disease profiles. We also include baseline data on metabolic conditions, such as hypertension, diabetes, hyperlipidemia, and fatty liver disease, which were accounted for during matching, given their growing link to liver cancer development [62,63].

This study has several limitations. The relatively short median follow-up of 19.1 months in the immune cell group, compared to the longer median follow-up in the control group, may underestimate late recurrence rates by disproportionately censoring events occurring later in the observation period, thereby overstating the therapy’s effectiveness [64]. This short follow-up duration is primarily due to the recent adoption of CIK therapy following the 2022 guidelines and its inclusion in national insurance, with a large proportion of CIK patients (40 out of 49) receiving their first immune cell injection in 2022 or later. While the data originates from a high-volume tertiary referral center and represents the first dataset collected after CIK therapy’s inclusion in national insurance coverage, the small sample size of the immune cell group limits generalizability and increases the risk of significant estimation errors, making it more likely to overestimate treatment benefits [65]. Furthermore, the high cost of CIK therapy restricts broader patient inclusion. The study’s focus on stage I and II patients and its retrospective design also introduce potential selection bias and confounding, limiting its applicability to wider HCC populations. We plan to conduct further research with larger cohorts and an extended follow-up in the near future to validate these results and investigate CIK therapy’s long-term efficacy.

## 5. Conclusions

In conclusion, adjuvant CIK cell immunotherapy significantly prolongs RFS in early-stage HCC patients and reduces AFP and PIVKA-II levels in high-risk subgroups with elevated post-treatment markers. Long-term outcomes such as late recurrence and overall survival remain unclear due to the short follow-up period. Further extended research is necessary to evaluate its long-term efficacy and explore broader applications in diverse patient populations.

## Figures and Tables

**Table 1:** Baseline characteristics of study population.

	Immune Cell Group (n = 49)	Control (Before Matching) (n = 2091)	*p* Value (Before Matching)	Control (After Matching) (n = 49)	*p* Value (After Matching)	SMD (After Matching)
Age, years	59.0 (53.0–66.0)	60.0 (53.0–67.0)	0.405	60.0 (48.0–65.0)	0.876	0.081
AST, IU/L	33.0 (26.0–42.0)	31.0 (24.0–46.5)	0.508	30.0 (23.0–38.0)	0.192	0.082
ALT, IU/L	31.0 (25.0–40.0)	29.0 (20.0–45.0)	0.393	29.0 (21.0–39.0)	0.296	0.042
T.bil, mg/dL	0.8 (0.6–0.9)	0.8 (0.6–1.1)	0.420	0.8 (0.6–1.0)	0.841	0.081
Albumin, g/dL	4.5 (4.3–4.8)	4.4 (4.0–4.6)	0.003	4.4 (4.1–4.7)	0.136	0.213
PT INR	1.0 (0.97–1.04)	1.0 (0.9–1.1)	0.622	1.0 (0.9–1.1)	0.284	0.034
PLT, ×10^3^/mm^3^	191.0 (152.0–237.0)	170.0 (129.0–214.0)	0.068	202.0 (163.0–236.0)	0.359	0.161
AFP, ng/mL	8.5 (2.8–100.3)	7.4 (3.2–45.6)	0.825	8.9 (2.9–48.9)	0.840	0.046
PIVKA-II, mAU/mL	79 (29.0–491.0)	37.0 (24.0–138.0)	0.021	80.0 (26.0–314.0)	0.696	0.285
Sex			0.589		0.814	0.095
Male	36 (73.5)	1626 (77.8)		38 (77.6)		
Female	13 (26.5)	465 (22.2)		11 (22.4)		
Hypertension			0.508		0.836	0.084
Yes	29 (59.2)	975 (46.6)		18 (36.7)		
No	20 (40.8)	1116 (53.4)		31 (63.3)		
Diabetes			0.097		1.000	0.048
Yes	11 (22.4)	730 (34.9)		12 (24.5)		
No	38 (77.6)	1361 (65.1)		37 (75.5)		
Dyslipidemia			0.451		1.000	0.000
Yes	6 (12.2)	364 (17.4)		6 (12.2)		
No	43 (87.8)	1727 (82.6)		43 (87.8)		
Fatty Liver			<0.001		0.809	0.098
Yes	12 (24.5)	167 (8.0)		10 (20.4)		
No	37 (75.5)	1924 (92.0)		39 (79.6)		
Liver Cirrhosis			0.263		0.839	0.082
Yes	28 (57.1)	1004 (48.0)		26 (53.1)		
No	21 (42.9)	1087 (52.0)		23 (46.9)		
HCC Cause			0.205		0.819	0.132
HBV HCV	40 (81.6)	1461 (69.9)		40 (81.6)		
	2 (4.1)	129 (6.2)		1 (2.0)		
Others	7 (14.3)	501 (24.0)		8 (16.3)		
Treatment			0.820		1.000	0.057
Resection	41 (83.7)	1701 (81.3)		42 (85.7)		
RFA	8 (16.3)	390 (18.7)		7 (14.3)		

Descriptive statistics for baseline characteristics of the immunotherapy and control groups are presented. Continuous variables are shown as median (IQR), while categorical variables are displayed as n (%). Mann–Whitney’s U-test was used for comparing continuous variables, and chi-square or Fisher’s exact test for binary variables. After matching, all *p*-values exceeded 0.05, and SMDs were below 0.30, indicating no significant differences in baseline characteristics between the two groups. Abbreviations: AST, aspartate aminotransferase; ALT, alanine aminotransferase; T.bil, total bilirubin; PT INR, prothrombin time international normalized ratio; PLT, platelet count; AFP, alpha-fetoprotein; PIVKA-II, protein induced by vitamin K absence-II; HCC, hepatocellular carcinoma; HBV, hepatitis B virus; HCV, hepatitis C virus; RFA, radiofrequency ablation; SMD, standardized mean difference; PSM, propensity-score matching; IQR, interquartile range.

**Table 2:** Predictors of RFS.

Variables	Univariable		Multivariable	
	HR (95% CI)	*p* Value	HR (95% CI)	*p* Value
Age > 59 vs. ≤59 years	0.84 (0.43–1.62)	0.600		
AST > 30 vs. ≤30 IU/L	1.32 (0.68–2.57)	0.406		
ALT > 30 vs. ≤30 IU/L	1.23 (0.64–2.36)	0.545		
T.bil > 0.80 vs. ≤0.80 mg/dL	0.98 (0.50–1.91)	0.951		
Albumin > 4.40 vs. ≤4.40 g/dL	1.33 (0.69–2.58)	0.390		
PT INR > 1.01 vs. ≤1.01	1.03 (0.53–1.97)	0.940		
PLT > 198 vs. ≤198 × 10^3^/mm^3^	1.01 (0.52–1.95)	0.976		
AFP > 8.69 vs. ≤8.69 ng/mL	1.46 (0.75–2.85)	0.270		
PIVKA-II > 79.50 vs. ≤79.50 mAU/mL	2.35 (1.19–4.65)	0.014	2.10 (1.05–4.21)	0.036
Male Sex	2.58 (1.00–6.65)	0.050	1.94 (0.74–5.09)	0.178
Hypertension	1.29 (0.67–2.49)	0.447		
Diabetes	1.51 (0.74–3.06)	0.258		
Dyslipidemia	0.90 (0.35–2.33)	0.829		
Fatty Liver	0.88 (0.40–1.93)	0.740		
Liver Cirrhosis	1.35 (0.68–2.67)	0.384		
HCC cause		0.314		
HBV	1 (Reference)			
HCV	1.14 (0.15–8.34)			
Others	1.90 (0.87–4.19)			
Resection vs. RFA	2.06 (0.72–5.85)	0.177		
Immune cell vs. Control	0.32 (0.15–0.71)	0.005	0.32 (0.15–0.71)	0.005

Factors associated with RFS based on univariable and multivariable analyses are shown. Continuous variables were categorized using their median values as cutoffs. Variables that were found to be significant (*p* < 0.05) in the univariable analysis were subsequently included in the multivariable analysis using a stepwise selection method. Abbreviations: RFS, recurrence-free survival; AST, aspartate aminotransferase; ALT, alanine aminotransferase; T.bil, total bilirubin; PT INR, prothrombin time international normalized ratio; PLT, platelet count; AFP, alpha-fetoprotein; PIVKA-II, protein induced by vitamin K absence-II; HCC, hepatocellular carcinoma; HBV, hepatitis B virus; HCV, hepatitis C virus; RFA, radiofrequency ablation; CI, confidence interval.

**Table 3:** Changes in tumor markers after immunotherapy (a) stratified by post-treatment AFP and (b) post-treatment PIVKA-II levels.

**(a) Stratification by Post-Treatment AFP Levels.**						
**Values**	**Post-Treatment AFP > 10 ng/mL**			**Post-Treatment AFP ≤ 10 ng/mL**		
	**Pre-CIK**	**Post-CIK**	***p*** **value**	**Pre-CIK**	**Post-CIK**	***p*** **value**
AFP (ng/mL)	15.3 (5.2–28.4)	1.3 (1.7–3.0)	0.002	2.8 (1.3–5.0)	2.1 (1.3–2.7)	0.552
PIVKA-II (mAU/mL)	26.0 (21.0–34.0)	26.0 (21.0–29.0)	0.307	31.5 (24.5–57.5)	29.5 (20.5–33.8)	0.097
**(b) Stratification by Post-Treatment PIVKA-II Levels.**						
**Values**	**Post-Treatment PIVKA-II > 40 mAU/mL**			**Post-Treatment PIVKA-II ≤ 40 mAU/mL**		
	**Pre-CIK**	**Post-CIK**	***p*** **value**	**Pre-CIK**	**Post-CIK**	***p*** **value**
AFP (ng/mL)	10.4 (1.3–15.3)	2.4 (1.7–7.9)	0.084	3.0 (2.0–5.7)	1.5 (1.3–2.3)	0.001
PIVKA-II (mAU/mL)	42.0 (27.0–74.0)	27.0 (23.0–33.0)	0.019	25.0 (21.0–30.3)	28.0 (17.8–33.0)	0.932

In the high tumor marker group (post-treatment AFP > 10 ng/mL), median AFP levels significantly decreased from 15.3 to 1.3 ng/mL (*p* = 0.002), while PIVKA-II levels showed no significant change (*p* = 0.307). In the low tumor marker group (post-treatment AFP ≤ 10 ng/mL), neither AFP (*p* = 0.552) nor PIVKA-II (*p* = 0.097) levels showed significant changes. For the high tumor marker group (post-treatment PIVKA-II > 40 mAU/mL), PIVKA-II levels significantly decreased from 42.0 to 27.0 mAU/mL (*p* = 0.019), whereas AFP levels showed a non-significant reduction (*p* = 0.084). In the low tumor marker group (post-treatment PIVKA-II ≤ 40 mAU/mL), AFP levels significantly decreased from 3.0 to 1.5 ng/mL (*p* = 0.001), but PIVKA-II levels remained unchanged (*p* = 0.932). Abbreviations: AFP, alpha-fetoprotein; PIVKA-II, protein induced by vitamin K absence-II; CIK, cytokine-induced killer cells.

## Data Availability

The data presented in this study are available on request from the corresponding author due to patient privacy concerns. Access to the data may be provided upon reasonable request and contingent upon ethical approval.

## Supplementary Materials

The following supporting information can be downloaded at: https://www.mdpi.com/article/10.3390/cancers17040566/s1, Table S1. Tumor characteristics of immune cell group and control group. Table S2. Details of anti-viral treatment in immune cell group and control group. Table S3. Comparison of cumulative RFS rates between immune cell group and control group. Table S4. Serum AFP and PIVKA-II levels after curative treatment and CIK cell therapy.

## Abbreviations

AFP: alpha-fetoprotein; AJCC, American Joint Committee on Cancer; ALT, alanine aminotransferase; AST, aspartate aminotransferase; CI, confidence interval; CIK, cytokine-induced killer; CT, computed tomography; CTL, cytotoxic T lymphocyte; HBV, hepatitis B virus; HCV, hepatitis C virus; HCC, hepatocellular carcinoma; HR, hazard ratio; IL, interleukin; IQR, interquartile range; IFN, interferon; KLCA, Korean Liver Cancer Association; MHC, major histocompatibility complex; MRI, magnetic resonance imaging; NA, not applicable; NCC, National Cancer Center; NK, natural killer; NSCLC, non-small cell lung cancer; OS, overall survival; PBMC, peripheral blood mononuclear cell; PLT, platelet count; PS, propensity score; PIVKA-II, protein induced by vitamin K antagonist-II; PT INR, prothrombin time international normalized ratio; RFA, radiofrequency ablation; RFS, recurrence-free survival; SMD, standardized mean difference; T.bil, total bilirubin; TCR, T-cell receptor.
