# Supplementary material for: Cytokine-Induced Killer Cell Immunotherapy Reduces Recurrence in Patients with Early-Stage Hepatocellular Carcinoma
Source: Cancers (Basel). 2025 Feb 7;17(4):566. doi: 10.3390/cancers17040566 (PMC11853259; doi:10.3390/cancers17040566)
Supplement: Supplementary file 1 [file cancers-17-00566-s001.zip › cancers-3400719-supplementary.pdf]

Table S1. Tumor characteristics of immune cell group and control group

	Immune Cell Group (n=41)	Control Group (n=35)	*P* value
Tumor Size	2.2 (1.4-3.7)	2.6 (1.8-4.2)	0.081
Tumor Number			1.000
<3	41 (100.0)	35 (100.0)	
Tumor Grade			1.000
≤2	39 (95.1)	34 (97.1)	
>2	2 (4.9)	1 (2.9)	
Gross Type			0.593
VN	2 (4.9)	0 (0.0)	
EN	15 (36.6)	11 (31.4)	
MC	19 (46.3)	21 (60.0)	
NP	3 (7.3)	2 (5.7)	
INF	2 (4.9)	1 (2.9)	
Tumor Necrosis			0.747
Present	15 (36.6)	15 (42.9)	
None	26 (63.4)	20 (57.1)	
Satellite Nodule			1.000
Present	1 (2.4)	1 (2.9)	
None	40 (97.6)	34 (97.1)	
Septum Formation			0.352
Present	24 (58.5)	25 (71.4)	
None	17 (41.5)	10 (28.6)	
Portal Vein Invasion			0.517
Present	8 (19.5)	4 (11.4)	
None	33 (80.5)	31 (88.6)	

Bile Duct Invasion			0.167
Present	4 (9.8)	0 (0.0)	
None	37 (90.2)	35 (100.0)	
Hepatic Vein Invasion			0.936
Present	0 (0.0)	1 (2.9)	
None	41 (100.0)	34 (97.1)	
Hepatic Artery Invasion			1.000
Present	0 (0.0)	0 (0.0)	
None	41 (100.0)	35 (100.0)	
Microvessel Invasion			1.000
Present	17 (41.5)	15 (42.9)	
None	24 (58.5)	20 (57.1)	

Descriptive statistics for tumor characteristics of the immunotherapy and control groups are presented. Continuous variables are shown as Median (IQR), while categorical variables are displayed as N (%). Mann-Whitney's U-test was used for comparing continuous variables, and a chi-square or Fisher's exact test for categorical variables. 8 patients from the immune cell group and 14 patients from the control group were excluded due to missing data. The Edmondson grading system was used for tumor grading, while the gross type classification followed the KLCA Guidelines.

Abbreviations: VN, Vaguely Nodular; EN, Expanding Nodular; MC, Multinodular Confluent; NP, Nodular with Perinodular Extension; INF, Infiltrative; IQR, Interquartile Range; KLCA, Korean Liver Cancer Association.

Table S2. Details of anti-viral treatment in immune cell group and control group

Hepatitis Type	Anti-Viral Treatment	Immune Cell Group (n=42)	Control Group (n=41)
HBV	Entecavir	12	16
HBV	Tenofovir Alafenamide	11	3
HBV	Tenofovir Disoproxil Fumarate	11	15
HBV	Lamivudine	0	1
HBV	Adefovir Dipivoxil	1	0
HBV	Non-treated	5	5
HCV	Pegylated Interferon α with Ribavirin	1	0
HCV	Non-treated	1	1

The table summarizes the use of anti-viral treatments in patients with HCC caused by hepatitis B or C in the immune cell (n=42) and control groups (n=41). Non-treated patients (6 each in immune cell group and control group) were identified, and reasons for non-treatment included undetectable HBV DNA, HBsAg conversion, or resolved infection.

Abbreviations: HBV, Hepatitis B Virus; HCV, Hepatitis C Virus; HCC, Hepatocellular Carcinoma.

Table S3. Comparison of cumulative RFS rates between immune cell group and control group

Outcome	Immune Cell Group (n=49)	Control Group (n=49)	*P* value
24 months	86.1 %	54.8 %	< 0.001
36 months	80.4 %	52.6 %	0.002
60 months	67.0 %	47.5 %	0.026
72 months	67.0 %	44.5 %	0.013
84 months	67.0 %	41.1 %	0.005

Cumulative RFS rates at 24, 36, 60, 72, and 84 months for the immune cell group and control group are shown. The p-values are derived from a one-sided z-test comparing the two groups at each time point. The results demonstrate a significantly higher RFS rate in the immune cell group compared to the control group, particularly in preventing early recurrence (within 2 years), as indicated by the 24-month cumulative RFS rate.

Abbreviations: RFS, Recurrence-Free Survival.

Table S4. Serum AFP and PIVKA-II levels after curative treatment and CIK cell therapy

Values	Curative Treatment			CIK Cell Therapy		
	**Before**	**After**	***P* value**	**Before**	**After**	***P* value**
AFP (ng/mL)	10.7 (3.6-112.5)	6.9 (2.8-42.1)	0.270	4.6 (1.7-14.6)	2.0 (1.3-2.9)	0.017
PIVKA-II (mAU/mL)	142.0 (38.5-575.5)	38.0 (23.0-80.5)	0.004	28.0 (23.0-53.5)	27.0 (21.0-33.0)	0.160

Descriptive statistics are shown for AFP and PIVKA-II values before/after curative treatment and immunotherapy. They are presented as Median (IQR). For comparisons. Mann-Whitney's U-tests were conducted. The specific comparisons made are between measurements taken before and after treatments to assess the changes in these biomarkers. The change in PIVKA-II levels before and after curative treatment, and in AFP levels before and after immunotherapy show a low p-value which indicates a statistically significant change between these time points.

Abbreviations: AFP, Alpha-Fetoprotein; PIVKA-II, Protein Induced by Vitamin K Absence-II; CIK, Cytokine-Induced Killer Cells; IQR, Interquartile Range.
